# MIMO-OFDM JSAC Waveform Design Based on Phase Perturbation and Hybrid Optimization

**DOI:** 10.3390/s25227010

**Published:** 2025-11-17

**Authors:** Zheming Guo, Baixiao Chen, Shuai Peng

**Affiliations:** National Key Laboratory of Radar Signal Processing, Xidian University, Xi’an 710071, China; zmguo163@163.com (Z.G.); shuaipeng_ss@163.com (S.P.)

**Keywords:** joint sensing and communication, waveform design, phase perturbation, hybrid optimization, multi-input multi-output

## Abstract

With the increasing sophistication of electromagnetic environments in modern combat platforms, joint sensing and communication (JSAC) technology has emerged as a critical research frontier. Among these, JSAC waveform design plays a crucial role, as it enables the simultaneous achievement of both sensing and communication functions using the same transmit waveform. This paper presents a novel waveform design for a multi-input multi-output (MIMO) JSAC system. The proposed design leverages orthogonal frequency division multiplexing (OFDM) to reduce signal interference through low cross-correlation characteristics. Linear frequency modulation (LFM) is used as the carrier waveform, effectively narrowing the main lobe width of the autocorrelation function. We introduce phase perturbation into binary phase shift keying (BPSK) signals to enhance waveform performance, formulating the resulting problem as a high-dimensional, non-convex optimization challenge. To address this, we propose a hybrid optimization algorithm QGPV combining a quantum genetic algorithm (QGA), quantum particle swarm optimization (QPSO), and variable neighborhood search (VNS). The simulation results demonstrate that the proposed algorithm achieves superior performance compared with several typical methods. Notably, the peak sidelobe level (PSL) can be suppressed to around −21 dB with five iterations, highlighting the efficiency of the optimization process. These results validate the effectiveness of the proposed approach, showing improved waveform characteristics with an acceptable trade-off in communication performance.

## 1. Introduction

The rapid proliferation of connected devices and the growing demand for bandwidth-intensive applications have drastically intensified competition for the limited spectral resources within the electromagnetic spectrum [1,2,3]. This escalating demand has rendered spectrum scarcity a critical bottleneck that constrains the development of modern sensing and communication technologies. To overcome this challenge, joint sensing and communication (JSAC) has emerged as a promising paradigm that enables efficient spectrum sharing and alleviates spectrum congestion [4,5,6]. By integrating sensing and communication functionalities into a unified framework, JSAC not only enhances spectrum utilization but also improves system-level resource allocation, thereby mitigating spectrum pressure. Moreover, JSAC offers additional benefits, including reduced hardware size and weight, lower manufacturing and maintenance costs, and improved energy efficiency [7,8]. With continuous technological advancements, JSAC is anticipated to play a pivotal role in diverse application domains—ranging from military operations to civilian services and intelligent transportation—ultimately fostering innovation in next-generation sensing and communication systems [9].

At present, two primary approaches are commonly adopted for designing JSAC waveforms [10]. The first approach embeds communication information into a sensing waveform through phase modulation [11], while the second approach directly employs a communication waveform for sensing purposes [12]. Although both strategies enable dual-functionality, they face significant challenges in achieving an effective trade-off between sensing accuracy and communication reliability, thereby motivating further research into optimized waveform design.

Orthogonal Frequency Division Multiplexing (OFDM) divides the available bandwidth into multiple orthogonal subcarriers, thereby achieving high spectral efficiency, strong robustness against multipath fading, and flexible resource allocation, and it has been widely applied in modern wireless communication systems [13]. In the context of JSAC, OFDM exhibits significant advantages by enabling simultaneous information transmission and target sensing within the same spectral resources. A considerable number of researchers have focused on exploring the application of OFDM in JSAC systems. In order to reduce the interference between sensing and communication, a robust OFDM waveform is proposed in [14], where the scattered communication signals from the target are utilized for sensing. In order to obtain super-resolution range and velocity estimates using OFDM, researchers have developed an automatic pairing approach for high-resolution range and velocity estimation, which further demonstrates the applicability of OFDM in JSAC systems [15]. In [16], researchers proposed an OFDM-based integrated radar and communication system architecture and further investigated the trade-offs among spectrum utilization, sensing accuracy, and communication reliability, thereby establishing a theoretical foundation for the design optimization of JSAC systems.

Recognized as a pivotal technology in modern communications, multiple-input multiple-output (MIMO) not only enhances capacity and reliability in communication systems but also provides critical capabilities for sensing functions, making it highly relevant for JSAC applications [17,18]. Reference [19] proposes a technique for combining MIMO and phased array systems, where each subarray transmits orthogonal waveforms. Reference [20] derives the Cramer–Rao bound and mutual information to achieve cooperation between MIMO radar and MIMO communication systems. It establishes a trade-off between the performance of the two systems and provides valuable insights for future research on system-level optimization strategies in JSAC systems. In [7], the joint design of the radar transmit code, radar receive filter, and communication system codebook for MIMO radar and communication coexistence is addressed.

In addition, reference [21] demonstrates the use of minimum shift keying (MSK) to modulate linear frequency modulated (LFM) signals, which are commonly used in radar applications. However, modulating information onto LFM signals reduces their Doppler tolerance. In [22], a method combining wireless sensing and communication is proposed, incorporating spectral sparse radar waveforms into the communication signal. Reference [23] suggests introducing phase perturbation to traditional communication signals to optimize their autocorrelation function. The communication receiver interprets insignificant phase perturbation as phase noise, allowing phase codes to be decoded accurately. In sensing processing, range compression is performed using known and optimized phase perturbation to achieve low-range sidelobes. Reference [24] investigates hybrid signals that integrate radar and communication information within the same radar pulse width through a series of short-duration phase changes. These phase variations appear as phase discretes in the LFM radar pulse and encode the information together with the radar signal. Reference [17] designs unimodular joint radar and communication (JRC) waveforms with low sidelobe levels by combining an LFM waveform and a BPSK waveform. In [25], binary information is embedded into a quadrantal waveform, and the sidelobe levels of the directional graph in the target direction are controlled using an optimization method. This modulation is equivalent to amplitude shift keying (ASK), where each pulse period transmits a bit of information. Reference [26] proposes a method to encode *L* bits of information (as a single base-$2^{L}$ symbol) into *Q* orthogonal waveforms, optimizing the phases of each waveform and the reference waveform in the target direction. The modulation of the signal is equivalent to phase shift keying (PSK), with each pulse period transmitting Q×L bits of information. In [27], PSK signals are embedded into frequency-hopping (FH) signals, allowing MIMO radar to function as a communication system.

Motivated by the above-mentioned studies, we first build an MIMO-OFDM JSAC system framework in this paper. More specifically, the use of OFDM significantly mitigates mutual interference among signals and ensures low cross-correlation, thereby improving waveform orthogonality. In this framework, using LFM as the signal carrier reduces the mainlobe width of the autocorrelation function, thereby enhancing the applicability of the signal. Furthermore, by combining BPSK with phase perturbations, a novel JSAC waveform is designed. Reducing the autocorrelation sidelobe level is essential for JSAC waveform design. To this end, we establish an optimization model for the peak sidelobe level (PSL) of the autocorrelation function of the JSAC signals, where the phase and phase perturbations are treated as optimization variables. The autocorrelation function expression is derived, and the PSL is adopted as the evaluation metric. This problem is formulated as a high-dimensional, non-convex optimization problem, which is known to be NP-hard [28]. Solving this problem using conventional algorithms is challenging. Intelligent optimization algorithms are well-suited to address high-dimensional, non-convex optimization problems directly. Reference [29] uses genetic algorithms (GA), artificial fish swarm algorithms (AFSA), and particle swarm optimization (PSO) to solve the optimal positioning problem in multi-station passive positioning systems. In [30], a hybrid intelligent dynamic optimization method integrating PSO and gradient-based optimization (GBO) is proposed for the optimal control of switched systems. To prevent premature convergence in PSO, paper [31] proposes an improved quantum-based PSO (QPSO) algorithm. In [32], a subroutine-based quantum GA (QGA) is proposed, with individuals encoded in independent registers, distinguishing it from previous approaches. It is worth noting that, based on QPSO, reference [33] incorporates the variable neighborhood search (VNS) algorithm. In each iteration step of QPSO, through the local search of VNS, it searches for the neighborhood of the current optimal solution and obtains a better solution, thereby further enhancing the effectiveness of the algorithm.

In this work, we propose a hybrid optimization algorithm QGPV that integrates QGA, QPSO and VNS for efficiently addressing the PSL of the autocorrelation function in the JSAC signals. In contrast to existing methods, we consider two stages to optimize the phase and phase perturbation. In the first step, QGA is employed to optimize the discrete phase. Specifically, since the BPSK phase is determined by a binary sequence carrying the communication information, which must remain unchanged, an additional binary sequence of equal length is introduced as the optimization variable. The optimized sequence is then linearly combined with the original communication sequence to generate the optimized phase, thereby completing the transformation from BPSK to QPSK. It is found that this process does not change the bit error rate (BER). In the second step, QPSO and VNS are used to optimize the phase perturbation, and its effect on the BER is analyzed. The results show that the BER slightly increases due to the addition of phase perturbation. Meanwhile, optimizing phase perturbation can significantly reduce the PSL of the autocorrelation function. Overall, the significant contributions of our work can be outlined as follows:

- We propose a novel JSAC scheme that integrates MIMO, OFDM, and LFM. In this framework, MIMO provides spatial diversity, OFDM suppresses signal cross-correlation, and LFM narrows the mainlobe of the autocorrelation function, thereby jointly enhancing system resolution, detection performance, and communication reliability.
- We design a new JSAC waveform by integrating BPSK with the phase perturbation and analyzing its autocorrelation characteristics.
- We formulate a hybrid optimization algorithm QGPV that combines QGA, QPSO, and VNS to effectively minimize the PSL of the waveform’s autocorrelation function. The phase and phase perturbation are optimized in separate steps, and the results demonstrate that the slight increase in BER is acceptable in exchange for a significant reduction in PSL.

The remainder of this paper is organized as follows: Section 2 presents the MIMO-OFDM JSAC system model and waveform design. Section 3 discusses the problem formulation and hybrid optimization. In Section 4, the effects of optimization and phase perturbation on the BER are investigated. Section 5 provides the simulation results. Finally, Section 6 concludes this paper.

## 2. JSAC System Model and Waveform Design

In this section, we present the JSAC system model and waveform design. The MIMO-OFDM architecture enables multiple-signal transmission, efficient spectrum utilization, and improved resolution. Furthermore, the waveform design incorporates phase modulation and phase perturbation to provide greater flexibility for subsequent PSL optimization, thereby enhancing both sensing and communication performance.

### 2.1. JSAC System Model

**Figure 1:**
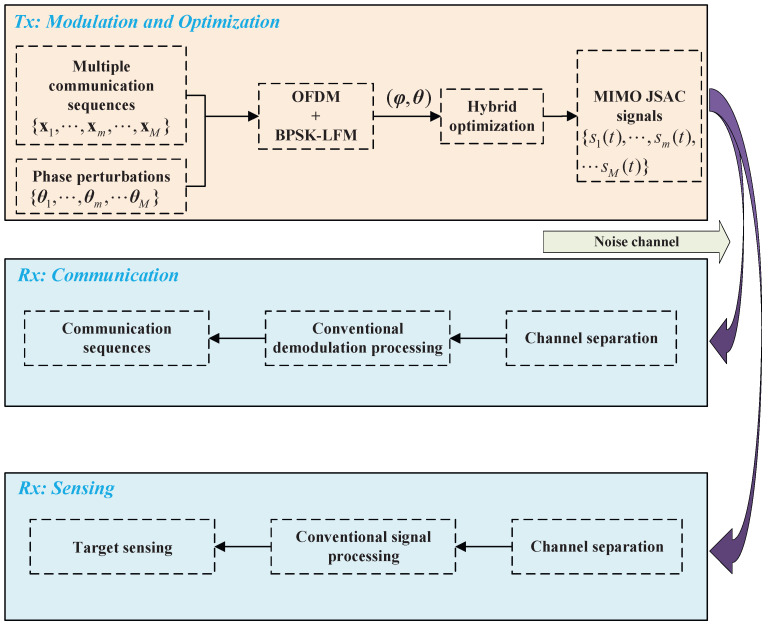
Block diagram of the proposed JSAC system.

Figure 1 illustrates a novel MIMO-OFDM JSAC system. At the transmitter, multiple communication sequences are assumed to be transmitted, each corresponding to a BPSK signal, where these sequences define the respective signal phases. Since the communication sequences to be transmitted are generally fixed and cannot be modified, additional binary sequences are established as optimization variables, each corresponding to one communication sequence. The optimized sequences are then combined with the original communication sequences to form the transmitted signal phases. However, optimizing only the signal phases often results in unsatisfactory suppression of the PSL of the autocorrelation function. To increase the optimization degrees of freedom, phase perturbations are incorporated into each signal, and a hybrid optimization approach is designed, which significantly improves the optimization results. In this framework, OFDM alleviates inter-symbol interference (ISI) and enhances spectral efficiency, while the use of LFM considerably narrows the mainlobe width of the autocorrelation function, thereby improving system applicability and enhancing resolution in sensing applications.

The optimization variables in the proposed system involve both discrete and continuous components, making the use of a single algorithm computationally impractical. To address this, we develop a two-stage hybrid optimization strategy: QGA is first employed to optimize the discrete phase, followed by QPSO and VNS to optimize the continuous phase perturbations, which effectively reduces the PSL of the autocorrelation function. The resulting JSAC signals are then ready for transmission over noisy channels.

On the communication receiving side, the communication sequences are decoded after channel separation. Meanwhile, conventional signal processing techniques, such as filtering and signal enhancement, are applied to improve the quality of the JSAC waveforms on the sensing receiving side.

It should be emphasized that this work is primarily concerned with the design of an MIMO-OFDM JSAC system and the optimization of its waveform at the transmitter, aiming to enhance both communication reliability and sensing accuracy. By jointly addressing system architecture and waveform optimization, the proposed approach provides a comprehensive framework for improving spectral efficiency and achieving more effective JSAC functionalities.

### 2.2. JSAC Waveform Design

Based on the proposed framework, we design an MIMO-OFDM JSAC waveform that incorporates OFDM, BPSK-LFM, and phase perturbation. In the MIMO architecture, multiple signals are transmitted simultaneously, thereby enhancing spatial diversity and system capacity. Generally, good orthogonality in JSAC waveforms is characterized by low autocorrelation sidelobe levels and low cross-correlation values among the transmitted signals. These properties ensure that a waveform is nearly independent of its time-shifted version and of any time-shifted version of other transmitted signals, thereby minimizing interference and enhancing system performance. To this end, OFDM is employed, where the orthogonality of subcarriers ensures low cross-correlation, efficient spectrum utilization, and robust resource allocation. Then, the waveform design further integrates LFM and BPSK modulation. Using LFM can effectively reduce the mainlobe width of the autocorrelation function, thereby improving range resolution and enhancing sensing accuracy. Meanwhile, BPSK is adopted to modulate communication information due to its robustness and implementation simplicity. To increase the degrees of freedom for subsequent optimization, phase perturbation is incorporated into the waveform, which facilitates effective suppression of the PSL and improves the trade-off between sensing accuracy and communication reliability. In this study, we first design the MIMO-OFDM JSAC waveform. Assuming that the JSAC system consists of *M* transmitting signals, the *m*-th transmitting signal can be expressed as:

(1) $$s_{m}\left(t\right)=\underset{n=1}{\sum^{N}\;}\;\mathrm{rect}\left\{\frac{t-\left(n-1\right)T_{b}}{T_{b}}\right\}\exp\left\{j[2\pi \left(f_{c}+f_{m}\right)t+\pi \mu t^{2}+\varphi_{m}\left(n\right)]\right\}$$

where *N* denotes the number of bits, and $T_{b}$ represents the bit duration. The signal duration is $T_{s}$ (i.e., $T_{s}=NT_{b}$). The parameter μ is the frequency modulation slope, and the signal bandwidth is given by $B=\mu T_{s}$. $f_{c}$ denotes the carrier frequency, and $f_{m}$ is the starting frequency, with m=1,2,…,M. Under normal circumstances, $f_{m}=\left(m-1\right)\Delta f$, where Δf is the frequency spacing between carrier frequencies. By properly setting Δf, orthogonality in the frequency domain can be ensured, thereby achieving favorable cross-correlation properties. rect(·) is the rectangular function, exp(·) denotes the exponential function, and j is the imaginary unit (i.e., $j^{2}=-1$). The phase of the *m*-th JSAC signal is expressed as $\varphi_{m}=\pi x_{m}$, where $\varphi_{m}=\left[\varphi_{m}\left(1\right),\ldots ,\varphi_{m}\left(n\right),\ldots ,\varphi_{m}\left(N\right)\right]$ and $x_{m}$ is a binary communication sequence defined as $x_{m}=\left[x_{m}\left(1\right),\ldots ,x_{m}\left(n\right),\ldots ,x_{m}\left(N\right)\right]$ with $x_{m}\left(n\right)\in \left\{0,1\right\}$.

From (1), it can be observed that the only directly optimizable variable is the phase sequence $\varphi_{m}$. However, optimizing the phase alone is insufficient to achieve effective suppression of the autocorrelation sidelobes, which often results in suboptimal waveform performance. To overcome this limitation, phase perturbation is employed, which significantly increases the degrees of freedom available for optimization. The inclusion of phase perturbations brings several advantages: it enables more flexible waveform adaptation, facilitates further reduction in the PSL, and improves the balance between sensing accuracy and communication reliability. In practical communication systems, such slight phase perturbations are generally interpreted as phase noise and thus have negligible impact on demodulation accuracy. This property ensures that the proposed method remains fully compatible with existing communication systems while being simple and efficient to implement. Consequently, the expression of $s_{m}\left(t\right)$ in (1) can be further written as follows:

(2) $$s_{m}\left(t\right)=\underset{n=1}{\sum^{N}}\;\mathrm{rect}\left\{\frac{t-\left(n-1\right)T_{b}}{T_{b}}\right\}\exp\left\{j\;\;\;[\;\;\;2\pi \left(f_{c}+f_{m}\right)t+\pi \mu t^{2}+\varphi_{m}\left(n\right)+\theta_{m}\left(n\right)]\right\}$$

where the phase perturbation $\theta_{m}=\left[\theta_{m}\left(1\right),\ldots ,\theta_{m}\left(n\right),\ldots ,\theta_{m}\left(N\right)\right]$ is randomly selected within the interval [−A,A]. The value of *A* depends on the modulation scheme; for instance, *A* should not exceed π in BPSK modulation and π/2 in QPSK modulation. This restriction arises because when *A* exceeds the phase decision threshold, the BER of the signal increases significantly, thereby reducing its practical applicability.

## 3. Problem Formulation and Optimization

In this section, based on the previously established JSAC system and signal model, we formulate the optimization problem of suppressing the autocorrelation sidelobe level of the JSAC waveform. First, the analytical expression of the autocorrelation function is derived, where high sidelobe levels are shown to adversely affect both sensing and communication performance. To address this issue, an optimization model is constructed with the PSL as the objective function, and a hybrid optimization algorithm QGPV is designed to effectively solve the problem.

According to (2) and reference [32], the autocorrelation function of the *m*-th JSAC signal can be calculated as follows:

(3) $$R_{m}\left(\tau ,\varphi_{m},\theta_{m}\right)=\left\{\begin{array}{c} \sum_{i=0}^{N-1}\mathrm{rect}\left(\frac{\tau -iT_{b}}{T_{b}}\right)\left[\begin{array}{c} \sum_{n_{2}=1}^{N-i}\int_{\left(n_{2}-1\right)T_{b}+\tau }^{\left(n_{2}+i\right)T_{b}}f(n_{2}+i,n_{2},t)dt+\\ \sum_{n_{2}=1}^{N-i-1}\int_{\left(n_{2}+i\right)T_{b}}^{n_{2}T_{b}+\tau }f(n_{2}+i+1,n_{2},t)dt \end{array}\right],\;0\leq \tau \leq NT_{b}\\ \sum_{i=-1}^{-N}\mathrm{rect}\left(\frac{\tau -iT_{b}}{T_{b}}\right)\left[\begin{array}{c} \sum_{n_{2}=1-i}^{N}\int_{\left(n_{2}-1\right)T_{b}+\tau }^{\left(n_{2}+i\right)T_{b}}f(n_{2}+i,n_{2},t)dt+\\ \sum_{n_{2}=-i}^{N}\int_{\left(n_{2}+i\right)T_{b}}^{n_{2}T_{b}+\tau }f(n_{2}+i+1,n_{2},t)dt \end{array}\right],\;-NT_{b}\leq \tau \leq 0 \end{array}\right.$$

where

(4) $$f\left(n_{1},n_{2},t\right)=e^{-j\pi \mu \tau^{2}}\cdot e^{j2\pi \tau (\mu t+f_{c}+f_{m})}\cdot e^{j[\varphi_{m}\left(n_{1}\right)-\varphi_{m}\left(n_{2}\right)]}\cdot e^{j[\theta_{m}\left(n_{1}\right)-\theta_{m}\left(n_{2}\right)]}$$

and

(5) $$\begin{array}{cc} \int_{a}^{b}f\left(n_{1},n_{2},t\right)dt= & e^{-j\pi \mu \tau^{2}}\cdot e^{j2\pi (f_{c}+f_{m})\tau }\cdot e^{j\left[\varphi_{m}\left(n_{1}\right)-\varphi_{m}\left(n_{2}\right)\right]}\cdot e^{j\left[\theta_{m}\left(n_{1}\right)-\theta_{m}\left(n_{2}\right)\right]}\\ \; & \cdot e^{j[\pi \mu \tau (a+b)]}\cdot \left(b-a\right)\cdot \sin c\left(\mu \tau \left(b-a\right)\right) \end{array}$$

where $\left[\varphi_{m}\left(n_{1}\right),\theta_{m}\left(n_{1}\right)\right]$ and $\left[\varphi_{m}\left(n_{2}\right),\theta_{m}\left(n_{2}\right)\right]$ represent the phases and the phase perturbations corresponding to the JSAC signals. sinc(·) is a sinc function.

For an ideal orthogonal signal, the autocorrelation function is the form of an impulse, whereas the cross-correlation function is identically zero, as presented in (6) and (7).

(6) $$R_{m}\left(s_{m},\tau \right)=\frac{1}{E}\int_{t}s_{m}\left(t\right)s_{m}^{*}\left(t+\tau \right)dt=\left\{\begin{array}{c} 1,\tau =0\\ 0,\tau \neq 0 \end{array}\right.,\;m=1,2,\ldots ,M$$

(7) $$C\left(s_{p},s_{q},\tau \right)=\int_{t}s_{p}\left(t\right)s_{q}^{*}\left(t+\tau \right)dt=0,\;p\neq q,\forall \tau \in R,\;p,\;q=1,2,\ldots ,M$$

where *E* is the energy of signal $s_{m}\left(t\right)$.

In practice, ideal orthogonal signals do not exist. As a result, the signals in this system only partially satisfy the conditions outlined in Equations (6) and (7). Moreover, since OFDM is employed to separate the spectrum of each signal, good orthogonality is maintained among the signals, which eliminates the need for additional optimization. Consequently, our focus shifts to minimizing the PSL of the autocorrelation function to further enhance signal quality.

It can be seen from (3) that the optimization of the autocorrelation function depends on both the discrete phase and the continuous phase perturbation. The phase varies in a discrete manner, similar to conventional BPSK modulation, whereas the phase perturbation is generated randomly within a given interval and changes continuously. This mixed-variable problem is inherently NP-hard and non-convex, which makes direct optimization with a single algorithm infeasible. To address this challenge, we adopt a hybrid optimization strategy that leverages the global search capability of population-based algorithms and the local search capability of VNS method, making it essential for optimizing efficient JSAC waveforms.

Through analytical derivation of the autocorrelation function, we formulate a hybrid optimization problem QGPV to suppress the autocorrelation PSL. The optimization strategy leverages the respective advantages of QGA, QPSO, and VNS, yielding an optimization result within an acceptable time frame. The optimization process follows a sequential, quantum-inspired and local search strategy. In the first stage, an additional binary sequence is randomly generated and optimized using QGA without altering the original communication data. According to the phase modulation principles of BPSK and QPSK, the optimized sequence is then combined with the transmitted binary communication sequence to obtain the optimized phase, thereby laying the foundation for subsequent PSL suppression. In the second stage, QPSO and VNS are employed to further optimize the autocorrelation function by adjusting the continuous phase perturbation. To improve convergence, VNS is incorporated into each iteration of QPSO, performing neighborhood-based searches around candidate solutions. This integration accelerates convergence, avoids premature stagnation, and enhances overall optimization performance. By leveraging the complementary strengths of QGA, QPSO, and VNS, the proposed hybrid algorithm achieves more effective PSL suppression. Based on the above analysis, we formulate the optimization problem as follows:

(8) $$\begin{array}{cc} \left(\varphi_{m-\mathrm{opt}},\theta_{m-\mathrm{opt}}\right) & =\underset{\varphi ,\theta }{\arg\min\max}\;{∥R_{m}\left(\varphi_{m},\theta_{m}\right)∥}_{\tau \neq 0}\\ s.t. & \varphi_{m}\left(1\right)=0,\;\theta_{m}\left(1\right)=0\\ \; & \left|\theta_{m}\left(n\right)\right|\leq A,\;n=2,\;\ldots ,\;N \end{array}$$

where $∥\cdot ∥$ denotes a vector norm, which can be defined either as the 2-norm or the infinity norm. When the 2-norm is adopted, the integrated sidelobe level (ISL) is suppressed. However, as this work focuses on the PSL, we adopt the infinity norm for $∥\cdot ∥$. In addition, the first symbol does not affect the amplitudes of the autocorrelation sidelobes; therefore, we set $\varphi_{m}\left(1\right)=0$ and $\theta_{m}\left(1\right)=0$. In this formulation, the phase $\varphi_{m}$ in (8) is treated as a discrete variable, whereas the phase perturbation $\theta_{m}$ is treated as a continuous variable.

From (8), it can be observed that both the objective function and the constraints are non-convex, making the problem NP-hard. Furthermore, $\varphi_{m}$ is a discrete variable, while $\theta_{m}$ is a continuous variable, and these two variables jointly determine the PSL of the autocorrelation function. Inspired by intelligent optimization algorithms, we propose a hybrid optimization algorithm QGPV, to address this problem. The proposed approach achieves satisfactory results within a limited computational time. It is designed as a two-stage process, where the output of the first stage serves as the input for the second stage.

As shown in Algorithm 1, the objective of the first stage is to optimize the phase $\varphi_{m}$ using QGA. It should be noted that the communication sequence $x_{m}$ is generally immutable, i.e., the communication information cannot be altered during the optimization process. To address this constraint, we generate a random binary sequence $y_{m}$ with the same length as $x_{m}$. Then, QGA iteratively optimizes $y_{m}$ by taking the PSL as the objective function. Once the termination criterion is satisfied, the optimized phase is obtained through the combination of $x_{m}$ and $y_{m}$, given by $\varphi_{m-\mathrm{opt}}=\pi \left(x_{m}+y_{m}/2\right)$. This optimized phase $\varphi_{m-\mathrm{opt}}$ is subsequently used as the input for the second stage optimization, where the phase perturbation $\theta_{m}$ is optimized using QPSO in conjunction with VNS. Specifically, during each iteration of QPSO, VNS is incorporated to refine the current solution. By switching among different neighborhood structures, VNS performs local searches around candidate solutions, thereby preventing premature convergence to local minima. This cooperative mechanism enhances the global exploration capability of QPSO. It also accelerates convergence and improves the robustness of the optimization process. After two stages of optimization, the PSL of the autocorrelation can be significantly suppressed, and the practicability of the JSAC signal can be improved.

Algorithm 2 illustrates the integration of VNS into each iteration of QPSO. As a local search strategy, VNS expands the search scope by employing multiple neighborhood structures, thereby improving the incumbent solution. These neighborhood structures enlarge the exploration space and increase the probability of escaping local optima. Specifically, during each iteration of QPSO, the neighborhood of the current best solution is explored. Through VNS, an improved solution is identified and subsequently used as the initial value for the next iteration. By embedding VNS into QPSO, the algorithm achieves enhanced global search capability and accelerated convergence.

**Algorithm 1:** Optimizing ($\varphi_{m}$,$\theta_{m}$) through QGPV

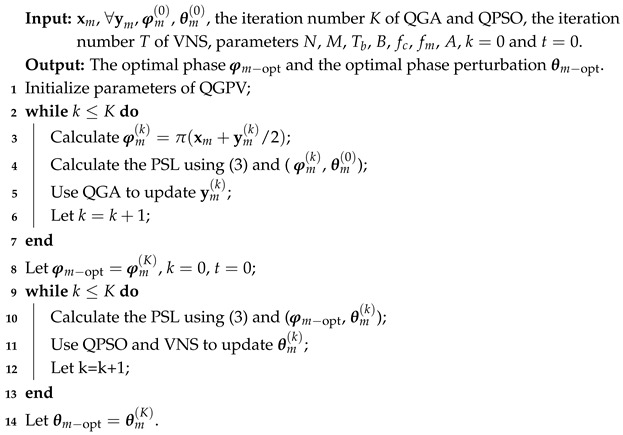



**Algorithm 2:** VNS-Assisted Update of $\theta_{m}$ in Iterations of QPSO

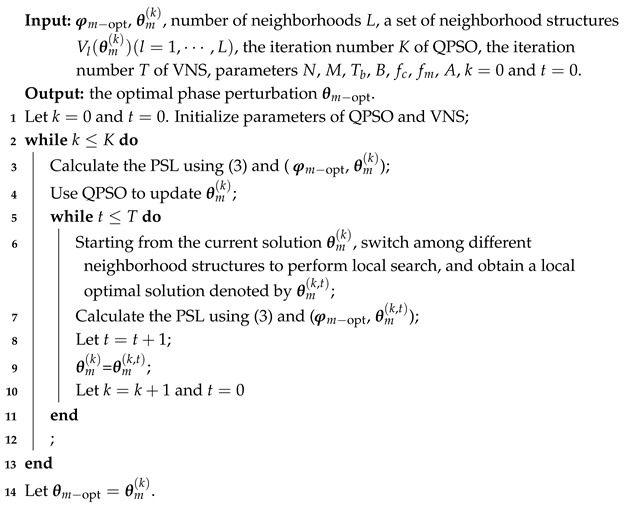



**Figure 2:**
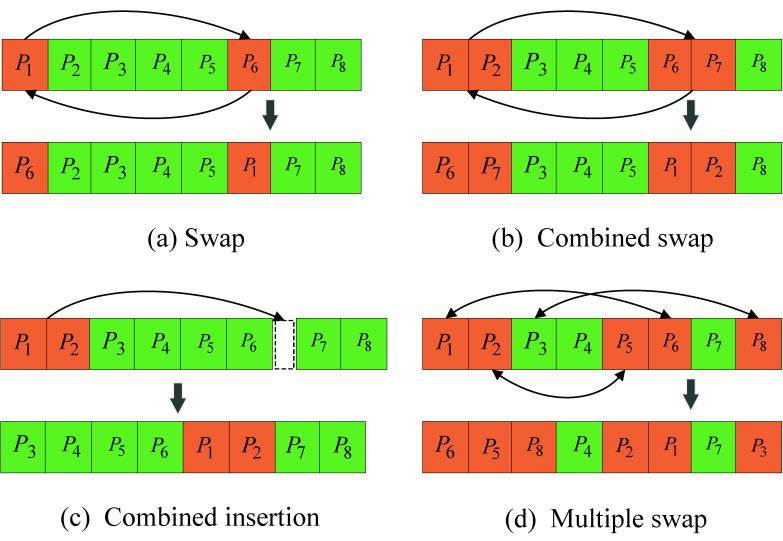
Illustration of four neighborhood structures.

Following the idea proposed in [33], we extend the method to a multi-point interchangeable neighborhood structure, as shown in Figure 2d. Figure 2 illustrates four neighborhood structures. According to the optimization problem formulated above, the current solution can be assumed to be a sequence of length 8. Figure 2a represents a neighborhood structure where two positions in the sequence are randomly selected and swapped. Figure 2b shows the case where two adjacent elements are randomly chosen and their positions are swapped. In Figure 2c, a gap is randomly selected and a pair of adjacent elements is inserted into this gap. For the established optimization model, the optimization variable is of considerable length, and thus local search based only on structures (a)–(c) is insufficient. To address this limitation, Figure 2d proposes a multi-point swap neighborhood structure. By setting an appropriate number of swapped positions, this structure further enhances the search capability.

**Figure 3:**
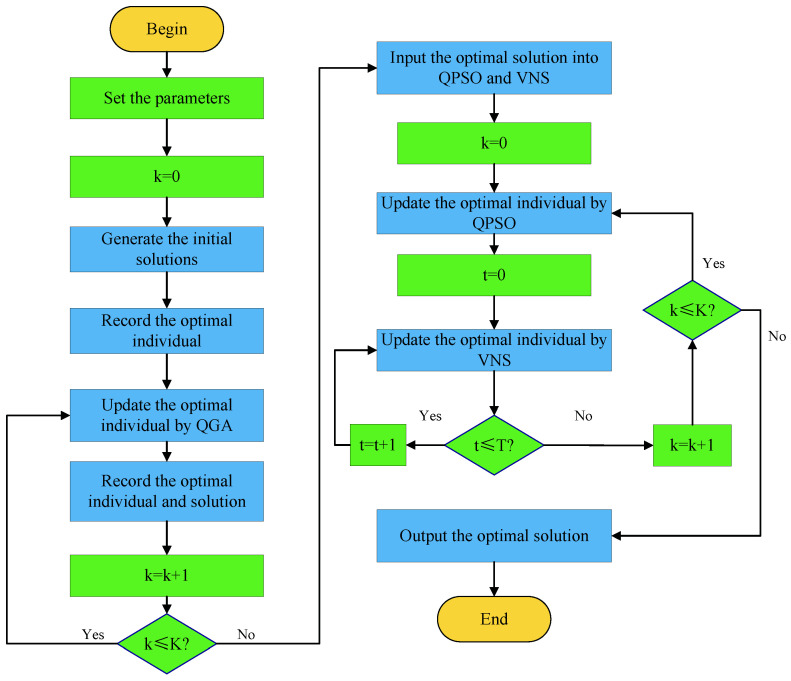
Flowchart for optimizing JSAC signal using QGPV.

The proposed hybrid optimization algorithm QGPV effectively integrates the strengths of QGA, QPSO, and VNS, thereby minimizing the PSL of the autocorrelation function, enhancing the practicability of the JSAC waveform, and improving the overall system performance. In the first stage, QGA is employed to optimize the phase, whereas in the second stage QPSO combined with VNS is utilized to refine the phase perturbation. This two-stage procedure ensures that the algorithm can obtain satisfactory results within a finite computational time. To provide a clearer understanding of the algorithmic procedure, Figure 3 presents the complete flowchart of the proposed method. This diagram also highlights the sequential interactions among its components. The flowchart not only illustrates the logic of the two-stage optimization process but also clarifies the function of each module, thereby making the algorithm easier to comprehend and more convenient for practical implementation.

In Figure 3, we illustrate the optimization of $y_{m}$ using QGA. After parameter initialization, an initial quantum population is obtained, i.e., $G_{0}=\eta_{0}1_{I\times J}$, where $1_{I\times J}$ denotes a matrix of dimension I×J with all entries equal to one. Here, *I* represents the individual length, *J* denotes the number of individuals, and $\eta_{0}$ represents the quantum phase. During the population initialization stage of a binary QGA, $\eta_{0}$ is typically set to π/4. This configuration ensures that each gene position in an individual has an equal probability of 50% to be randomly selected as either 0 or 1.

Then, we generate a matrix R of random numbers to simulate the quantum state collapse, where each element follows a uniform distribution in the interval [0,1],that is $r_{ij}∼U\left(0,1\right)$. By comparing the values of each element in $G_{0}$ and R, we obtain the binary population $G_{b}$. For each element $g_{ij}$ in $G_{b}$, it can be calculated by

(9) $$g_{ij}=\left\{\begin{array}{cc} 1, & \mathrm{if}r_{ij}>\cos^{2}\left(\eta_{0}\right)\\ 0, & \mathrm{if}r_{ij}\leq \cos^{2}\left(\eta_{0}\right) \end{array}\right.$$

Furthermore, the optimal result is saved by calculating the PSL and determining the maximum number of iterations. If the termination condition is not met, full interference crossover and quantum gate rotation mutation are performed. According to Reference [28], the quantum rotation angle and direction adjustment strategy of the quantum rotation gate mutation is summarized in Table 1.

As presented in Table 1, $g_{ij}$ indicates the binary value (0 or 1) of each gene position in the current individual, $b_{i}$ denotes the optimal individual, the fit(·) evaluates the fitness of both the current and optimal individuals, i.e., $g_{j}$ and b. The quantum rotation angle is $\Delta \eta_{ij}$, and $e(\alpha_{ij}\beta_{ij})$ specifies the rotation direction under various conditions. The parameters $\alpha_{ij}$ and $\beta_{ij}$ are two complex numbers representing the probability amplitudes of the corresponding states at each gene position of an individual, satisfying ${\alpha_{ij}}^{2}+{\beta_{ij}}^{2}=1$. Moreover, ${\alpha_{ij}}^{2}$ and ${\beta_{ij}}^{2}$ denote the probabilities that each gene position of an individual is in state 0 or state 1, respectively.

In this study, the phase optimization is performed using QGA. Based on the algorithm design described above, the proposed method can be regarded as a binary QGA. Therefore, Table 1 is modified and simplified to facilitate computation. In Table 1, b represents the current optimal solution, as defined in Reference [28]. This indicates that the rotation direction is directed only toward the optimal solution of the current generation. However, such a strategy may slow down the convergence speed. To overcome this limitation, a hybrid control strategy is designed. A control parameter *w* is defined to balance the rotation direction. During the update process, an individual does not always rotate from the the current optimal solution. Instead, it rotates from the global optimal solution with a probability of *w*. In this paper, *w* is set to 0.3. This design achieves a good balance between convergence and diversity. It ensures stable convergence toward the optimal direction, while the incorporation of the global optimal solution enhances population diversity and enables the algorithm to escape from local optima. Specifically, a random number between 0 and 1 is generated for each individual update. If this random number is greater than *w*, b in Table 1 represents global optimal solution; otherwise, it represents the current optimal solution.

For the computation of $e(\alpha_{ij}\beta_{ij})$ in Table 1, the rotation angle $\eta_{ij}$ is used to replace $\alpha_{ij}$ and $\beta_{ij}$, that is, a single quantum bit is represented by $\eta_{ij}$. According to Reference [28], a quantum bit (i.e., a gene position of an individual) can be expressed as $\alpha_{ij}|0\rangle +\beta_{ij}|1\rangle$. Equivalently, it can be represented by the rotation angle $\eta_{ij}$, i.e., $\cos\left(\eta_{ij}\right)|0\rangle +\sin\left(\eta_{ij}\right)|1\rangle$. In our model, the probabilities of a quantum bit being measured in states 0 and 1 are denoted by $\cos^{2}\left(\eta_{ij}\right)$ and $\sin^{2}\left(\eta_{ij}\right)$, respectively. The rotation angle $\eta_{ij}$ is constrained within the range of [0,π/2], since $\eta_{ij}$ in the interval [π/2,π] and its symmetric counterpart in [0,π/2] produce identical measurement probabilities. Therefore, Table 1 can be further simplified. We only need to consider the case where $\alpha_{ij}\beta_{ij}>0$, and the value of $e(\alpha_{ij}\beta_{ij})$ is determined by comparing the current individual with the optimal individual as well as their fitness values. For example, when $g_{ij}=1$, $b_{ij}$, and $\mathrm{fit}\left(g_{j}\right)\geq \mathrm{fit}\left(b\right)$, the lookup in Table 1 yields $e(\alpha_{ij}\beta_{ij})=1$. Consequently, the rotation angle is $\Delta \eta_{ij}e(\alpha_{ij}\beta_{ij})$, and the parameter $\eta_{ij}$ is updated using the expression $\eta_{ij}=\eta_{ij}+\Delta \eta_{ij}e(\alpha_{ij}\beta_{ij})$. Subsequently, the parameter $\eta_{0}$ in (9) is replaced by $\eta_{ij}$, and (9) is recalculated to update the population. After a finite number of iterations, the algorithm converges to an optimal solution within a finite time, thereby completing the phase optimization using the QGA.

After phase optimization by QGA, the phase perturbation is further optimized by QPSO and VNS. This optimization process is divided into two steps: particle movement and swarm update. According to the learning mode presented in reference [28], in the *k*-th iteration, the PSL of each individual in the population $H^{\left(k\right)}$ is calculated. Let $h_{best}$ denote the individual corresponding to the optimal result. The population $H^{\left(k\right)}$ is then reordered based on the size of the PSL values. The reordered population is denoted as $H_{s}^{\left(k\right)}$. Reference [34] indicates that the convergence of particles is guided by the attractor $Z^{\left(k\right)}$, which represents a stochastic combination of the current optimal $H_{s}^{\left(k\right)}$ and the global optimal solution $h_{best}$. This inherent randomness enables QPSO to perform global exploration effectively. As a result, particles evolve dynamically within promising regions, greatly improving the algorithm’s global search capability and convergence efficiency. The attractor $Z^{\left(k\right)}$ can be expressed as follows:

(10) $$Z^{\left(k\right)}=\varepsilon H_{s}^{\left(k\right)}+\left(1-\varepsilon \right)h_{best}1$$

where ε is a random number evenly distributed over the interval (0,1), 1 is an all-one matrix with the same dimension as $H_{s}^{\left(k\right)}$.The swarm update of QPSO can be obtained as follows:

(11) $$H_{s}^{\left(k+1\right)}=Z^{\left(k\right)}+\alpha \left|\sigma 1-H_{s}^{\left(k\right)}\right|\cdot \ln\left(\frac{1}{u}\right)$$

where α is called the expansion factor, which is generally set to 0.5 [28]. $\sigma =\frac{\sum_{i=1}^{I}h_{i}}{J}$, *I* stands for individual length and *J* stands for the number of individuals. *u* is a random number evenly distributed over the interval (0,1).

In each iteration of QPSO, a current solution is first obtained and then optimized by VNS, which conducts a local search to identify a locally optimal solution. This locally optimal solution is subsequently used as the input for the next iteration, thereby enhancing the overall search process. The detailed iterative procedure has already been provided in Algorithm 2, and is therefore not repeated here.

## 4. Analysis of BER

In JSAC systems, BER serves as a crucial performance metric and plays an important role in evaluating JSAC signal performance. This section first investigates the impact of transitioning from BPSK to QPSK on BER. It then examines the variations in BER when the JSAC signal undergoes phase perturbation, which is categorized into two types: phase perturbation with a uniform distribution and phase perturbation with a fixed value.

In modern communication modulation systems, BER is typically influenced by the minimum euclidean distance $d_{\min}$ between signal constellations and the noise power spectral density $N_{0}$ [35]. The parameter $d_{\min}$ plays a key role in determining the ability to distinguish between different signal states, while $N_{0}$ governs the level of interference that can corrupt the transmitted signal. Both factors collectively contribute to the error performance of the system.

Moreover, as indicated in reference [36], replacing the carrier of a QPSK signal with a LFM signal does not affect BER. Consequently, BER for a classic QPSK signal remains as follows:

(12) $$P_{e}=Q\left(\frac{d_{\min}}{\sqrt{N_{0}}}\right)$$

where $d_{\min}$ is the the minimum euclidean distance. The noise power spectral density is denoted by $N_{0}$, and $Q\left(x\right)=\frac{1}{\sqrt{2\pi }}\int_{x}^{\infty }e^{-t^{2}/2}dt$.

As outlined in Algorithm 1 of the optimization process, the method proposed in this paper maps the bit “0” in the BPSK sequence to phases 0 and π/2, and the bit “1” to phases π and 3π/2. This phase mapping effectively completes the transition from BPSK to QPSK, enabling improved signal modulation and enhanced communication performance.

We further examine the placement of these symbolic points in the complex plane. Specifically, bit “0” is mapped to points $a_{0}^{\left(0\right)}=1$ and $a_{0}^{\left(\pi /2\right)}=i$, while bit “1” is mapped to points $a_{0}^{\left(\pi \right)}=-1$ and $a_{0}^{3\pi /2}=-i$. On the complex plane, the coordinates of these points are expressed as follows:

(13) $$a_{0}=\left\{\begin{array}{cc} \;1, & \left(1,0\right)\\ \;i, & \left(0,1\right)\\ -1, & \left(-1,0\right)\\ -i, & \left(0,-1\right) \end{array}\right.$$

Next, we calculate the euclidean distance between these symbolic points

(14) $$d=\left\{\begin{array}{c} \sqrt{{\left(1-0\right)}^{2}+{\left(0-1\right)}^{2}}=\sqrt{2},\;1\to i\\ \sqrt{{\left(1+1\right)}^{2}+{\left(0-0\right)}^{2}}=2,\;1\to -1\\ \;\sqrt{{\left(1-0\right)}^{2}+{\left(0+1\right)}^{2}}=\sqrt{2},\;1\to -i\\ \;\sqrt{{\left(0+1\right)}^{2}+{\left(1-0\right)}^{2}}=\sqrt{2},\;i\to -1\\ \sqrt{{\left(0-0\right)}^{2}+{\left(1+1\right)}^{2}}=2,\;i\to -i\\ \;\sqrt{{\left(1-0\right)}^{2}+{\left(0+1\right)}^{2}}=\sqrt{2},\;-1\to -i \end{array}\right.$$

From the above calculation, it is evident that the minimum euclidean distance between any two adjacent symbols is $d_{\min}=\sqrt{2}$, independent of the phase selection corresponding to either bit “0” or bit “1”. The mapping rule primarily alters the bit-to-symbol correspondence, without impacting the geometric positioning of the symbols in the complex plane. As a result, the relative distances between adjacent symbols remain unchanged, preserving the overall structure of the modulation scheme.

Next, we further investigate the variation in BER due to phase perturbation. Specifically, we analyze the impact of different types of phase perturbation—such as uniform and fixed phase perturbation—on BER, providing insights into the robustness of the modulation scheme under various conditions.

Based on the results discussed above and as referenced in [35], BER of JSAC signal can be expressed as follows:

(15) $$P_{e}=2Q\left(\sqrt{2\gamma_{b}}\right)\left(1-\frac{1}{2}Q\left(\sqrt{2\gamma_{b}}\right)\right)$$

where $\gamma_{b}=\frac{E_{b}}{N_{0}}$ represents the ratio of bit energy to noise power spectral density.

After introducing phase perturbation, it can be observed from the previous discussion that the phase perturbation interval is [−A,A]. The total phase offset of the received signal is expressed as $\phi_{m}=\varphi_{m}+\theta_{m}$. It is evident that the phase perturbation influences the effective signal-to-noise ratio (SNR), which in turn affects the symbol decision process, ultimately leading to an increase in BER. For a fixed phase perturbation $\theta_{m}$, the effective SNR can be written as follows:

(16) $$\gamma_{b,\mathrm{eff}}=\gamma_{b}\cos^{2}\left(\theta_{m}\right)$$

By substituting (16) into (15), the BER under fixed phase perturbation can be derived as follows:

(17) $$P_{\left.e\right|\theta_{m}}=2Q\left(\sqrt{2\gamma_{b}}\cdot \cos\left(\theta_{m}\right)\right)\cdot \left(1-\frac{1}{2}Q\left(\sqrt{2\gamma_{b}}\cdot \cos\left(\theta_{m}\right)\right)\right)$$

Then, assuming that the phase perturbation $\theta_{m}$ follows a uniform distribution, its probability density function is:

(18) $$f_{\theta }\left(\theta_{m}\right)=\frac{1}{2A},\;\theta_{m}\in \left[-A,A\right]$$

Since the phase perturbation is evenly distributed, we need to integrate all possible perturbation angles to obtain the overall BER, which can be expressed as follows:

(19) $$P_{e_{1}}=\int_{-A}^{A}P_{e}\left(\theta_{m}\right)\cdot f_{\theta }\left(\theta_{m}\right)d\theta_{m}$$

Combining (17)–(19), BER after adding the phase perturbation can be written as follows:

(20) $$P_{e_{1}}=\frac{1}{2A}\int_{-A}^{A}2Q\left(\sqrt{2\gamma_{b}}\cdot \cos\left(\theta_{m}\right)\right)\cdot \left(1-\frac{1}{2}Q\left(\sqrt{2\gamma_{b}}\cdot \cos\left(\theta_{m}\right)\right)\right)d\theta_{m}$$

Moreover, in the case of high SNR, the value of Q(·) is usually small, so the quadratic term can be ignored, and cos(·) is even function; so, the $P_{e1}$ is finally written as follows:

(21) $$P_{e_{1}}\approx \frac{2}{A}\int_{0}^{A}Q\left(\sqrt{2\gamma_{b}}\cdot \cos\left(\theta_{m}\right)\right)d\theta_{m}$$

When the phase perturbation is a fixed value, (21) can be written as follows:

(22) $$P_{e_{1}}\left(\theta_{m}\right)=2Q\left(\sqrt{2\gamma_{b}}\cos\left(\theta_{m}\right)\right)$$

## 5. Simulation Results

All experiments were conducted on a computer equipped with an Intel(R) Core(TM) Ultra9-185H CPU @ 2.90 GHz and 32.0 GB of memory. The development environment was MATLAB R2023a. To evaluate the performance of the proposed algorithm QGPV, we performed simulation experiments and compared them with QPSO, QGA, QGA-QPSO, and QPSO-VNS. The simulation parameters were configured as follows: (i) signal parameters: the number of signals was set to M=4, the binary sequence length to N=64, the bandwidth is B=0.5 MHz, the bit duration $T_{b}=10\;\;\;\;\mu \;\;\;s$, $f_{c}=1$ MHz, Δf=1 MHz, A=π/10. (ii) QGPV parameters: K=5, T=1500, L=4, the number of population J=100, the quantum rotation angle is 0.01π and the control attractor is 0.5.

**Figure 4:**
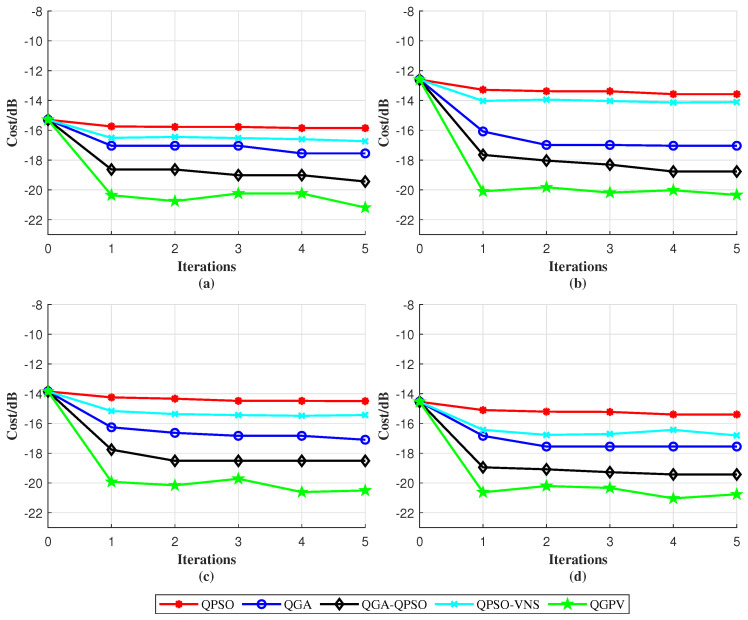
Optimization comparison of QPSO, QGA, QGA-QPSO, QPSO-VNS, and QGPV on (**a**) signal 1, (**b**) signal 2, (**c**) signal 3 and (**d**) signal 4.

The iteration comparison curves of the five algorithms are shown in Figure 4. Under the condition of five iterations, the optimization results of QPSO, QGA, QGA-QPSO, and QPSO-VNS are not satisfactory, mainly due to their slow convergence speed, premature stagnation, and poor sidelobe suppression performance. In contrast, QGPV converges faster and achieves a lower PSL level, showing a clear advantage over the other four algorithms. It achieves a PSL of approximately −21 dB, which is about 2 dB better than the best result obtained by the other four algorithms. This indicates that QGPV possesses stronger global search capability and better convergence stability.

In addition, when the problem scale is relatively small, i.e., the parameter N=[4,8,16], the global optimal solutions can be obtained using solvers such as BARON (25.3.19), Gurobi (11.0), FICO (8.11.2), and the MATLAB Optimization Toolbox (R2023a). As shown in Table 2, a comparison between the QGPV optimization results and the global optimal solutions is presented. The results indicate that QGPV achieves performance close to the global optimum in all cases, demonstrating its effectiveness and robustness. However, as the parameter *N* increases, the dimensionality and search space expand rapidly. Consequently, the solvers are unable to find the global optimum within a reasonable computation time. In fact, when N>16 and increases exponentially, their computational cost far exceeds that of QGPV. In contrast, QGPV achieves an effective trade-off between computational efficiency and optimization performance, obtaining a low PSL with significantly reduced computation time. Therefore, it is more suitable for large-scale signal design problems.

**Figure 5:**
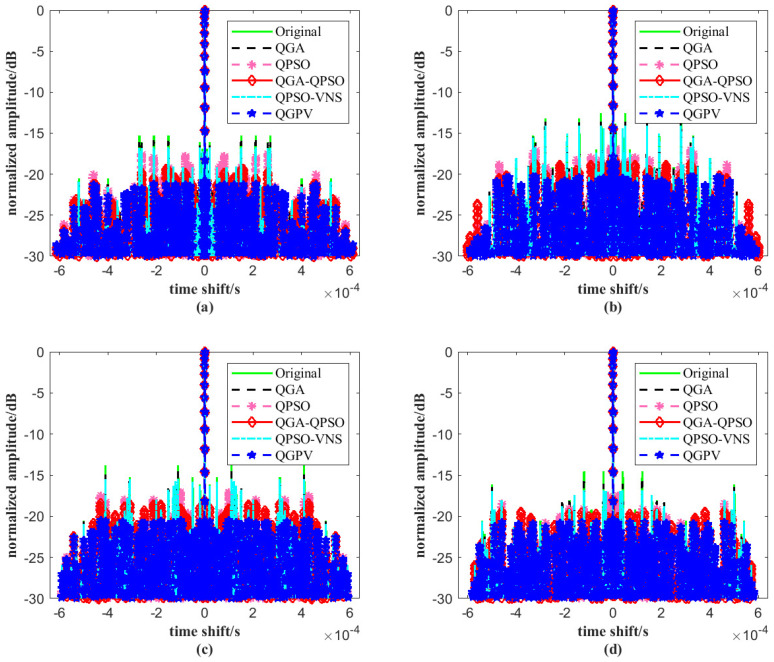
Autocorrelation values of the JSAC signal among Original, QPSO, QGA, QGA-QPSO, QPSO-VNS, and QGPV under *M* = 4 and *N* = 64 on (**a**) signal 1, (**b**) signal 2, (**c**) signal 3 and (**d**) signal 4.

Figure 5 illustrates the autocorrelation functions of the JSAC signal optimized by the four algorithms, with the parameters set to *M* = 4 and *N* = 64. As shown in Figure 5, under the same conditions, the autocorrelation likelihood values of the JSAC signal optimized by QGPV remain consistently lower than those obtained by the other four algorithms. This indicates that the proposed algorithm achieves significantly higher convergence accuracy in optimizing the autocorrelation characteristics of the JSAC signal and outperforms the other three algorithms in terms of performance. Such improvement is crucial for enhancing the overall performance of the JSAC system.

Table 3 presents the PSL optimization results of four signal autocorrelation functions obtained by five algorithms under five iterations. The results show that the proposed QGPV algorithm achieves the best overall performance. For all signals, QGPV produces lower PSL values than the other four algorithms. This demonstrates its strong optimization capability and robustness. These findings also confirm that QGPV maintains excellent sidelobe suppression performance even with a limited number of iterations.

**Figure 6:**
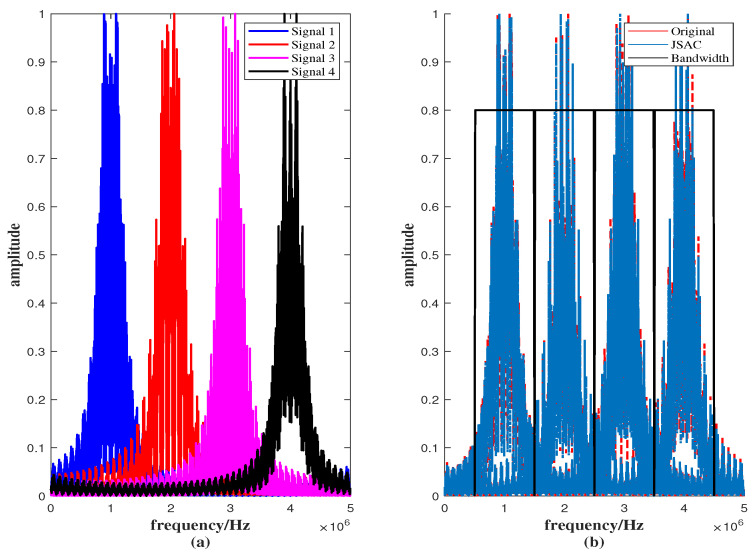
The signal spectra under *M* = 4, *N* = 64 and f=[1,2,3,4]MHz on (**a**) signals of the Equation (1), (**b**) JSAC signals.

According to Equation (1), the spectral characteristics of the four signals are analyzed. From Figure 6a, it can be observed that the spectra of the four signals are well separated, with no significant overlap between their frequency bands. The normalized amplitude in the overlapping region is approximately 0.04, corresponding to about −28 dB. Such a small overlap indicates that the signals are effectively non-overlapping and that mutual interference can be neglected. These results verify the effectiveness of the spectral separation design. As shown in Figure 6b, after introducing phase perturbation, the spectrum of the JSAC signal remains consistent with that of the original signal, indicating good structural stability. The normalized amplitude in the overlapping region between the two JSAC signal spectra is approximately 0.03, which corresponds to about −30 dB. This very small overlap implies that the interference effect is negligible and does not cause any noticeable degradation in system performance. Overall, the spectral separation design exhibits strong capability in interference suppression and signal isolation.

**Figure 7:**
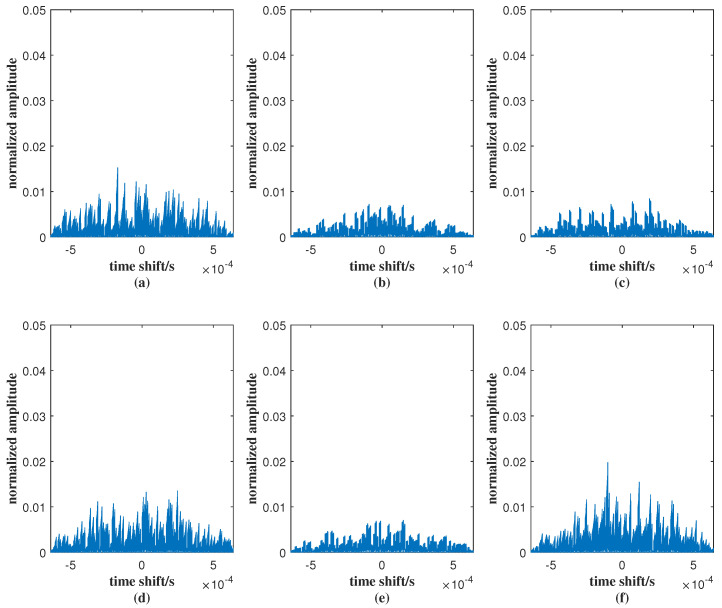
Cross-correlation values of the JSAC signal under *M* = 4 and *N* = 64 on (**a**) between signal 1 and 2, (**b**) between signal 1 and 3, (**c**) between signal 1 and 4, (**d**) between signal 2 and 3, (**e**) between signal 2 and 4, (**f**) between signal 3 and 4.

In Figure 7, the cross-correlation peak values of (a)–(f) are 0.015, 0.010, 0.006, 0.016, 0.007, and 0.016, respectively. These values are all close to zero, indicating that the correlations between different signals are very low. Such low cross-correlation levels demonstrate the excellent orthogonality of the designed signals, which effectively minimizes mutual interference and ensures good signal separation performance.

**Figure 8:**
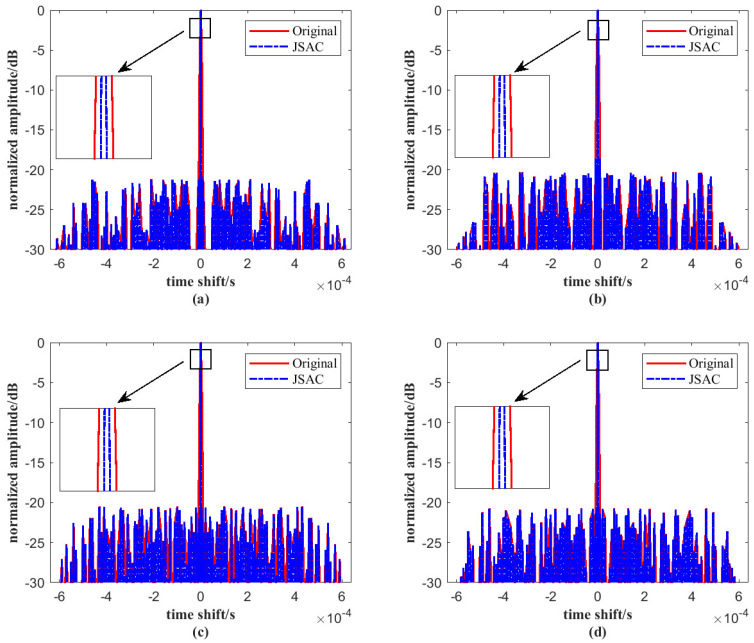
Comparison for the main lobe of autocorrelation function under *M* = 4 and *N* = 64 on (**a**) signal 1, (**b**) signal 2, (**c**) signal 3, (**d**) signal 4.

Figure 8 compares the main lobes of the original signal and the JSAC signal. As shown, incorporating the LFM signal into the JSAC system leads to a narrower autocorrelation main lobe. In addition, the 3-dB bandwidth is locally enlarged. It can be observed that the bandwidth becomes noticeably narrower, which further verifies the effectiveness of the proposed method. This improvement is attributed to the large time–bandwidth product of the LFM signal. As the time–bandwidth product increases, the main lobe of the autocorrelation function becomes narrower, which enhances angular resolution and improves resistance to interference. The narrowing of the main lobe also contributes to higher detection accuracy and better anti-interference capability. Therefore, the use of LFM signals in the JSAC system offers significant advantages, particularly in terms of resolution and robustness in complex signal environments.

**Figure 9:**
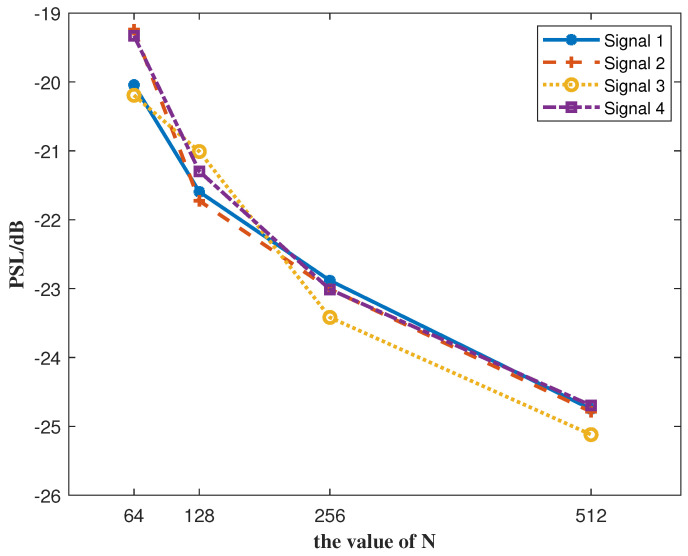
Performance of QGPV in optimizing four signals for different values of *N*.

To further demonstrate the effectiveness of the proposed algorithm, we evaluated the optimization performance of QGPV for four signals with different sequence lengths *N*. As shown in Figure 9, the optimization results improve as *N* increases, while all other parameters remain unchanged. When N=512, the PSL can be suppressed to approximately −25 dB. This trend indicates that larger sequence lengths provide more degrees of freedom for optimization, thereby enhancing the suppression of autocorrelation sidelobes. The results confirm that the proposed algorithm maintains robust performance under varying sequence lengths and achieves superior sidelobe suppression, which is crucial for improving the sensing and communication capabilities of JSAC systems.

As mentioned above, the integration of VNS into each iteration of QPSO significantly enhances the optimization performance, at the cost of a slight increase in computational time. However, this additional cost remains acceptable.

As shown in Table 4, the results of 100 Monte Carlo experiments demonstrate the average optimization results and running time for each algorithm. It can be observed that the proposed algorithm QGPV achieves the best optimization result (−20.5 dB), although with a marginally longer execution time, approximately 0.10 s. The runtime increases only slightly—approximately five times that of QPSO, ten times that of QGA, three times that of QGA-QPSO, and nearly the same as that of QPSO-VNS. Importantly, the increase in computational cost is relatively small compared with the gain in sidelobe suppression, and the total running time remains within a practically feasible range. In real-world applications, such a trade-off is highly desirable, as the slight increase in time does not affect signal processing requirements but leads to a substantial improvement in overall system performance.

According to the average running time of QGPV in Table 4, the running time of all algorithms was further restricted to 0.1 s. The average PSL optimization results and the mean iteration counts were calculated from 100 Monte Carlo experiments. As shown in Table 5, under identical running time conditions, the PSL optimization result of QPSO is −14.9 dB with an average of 28 iterations; QGA achieves −18.0 dB with 43 iterations on average; QGA-QPSO achieves −19.2 dB with 37 iterations; and QPSO-VNS achieves −15.6 dB with 5 iterations. The proposed QGPV algorithm still performs the best, achieving a PSL optimization result of −20.3 dB with an average of 5 iterations. These results further confirm the high efficiency of the QGPV.

Table 6 presents a comparison of the computational complexities of the algorithms. In this table, $I_{g}$ and $I_{p}$ denote the lengths of individuals, $J_{g}$ and $J_{p}$ represent the population sizes, and $K_{g}$ and $K_{p}$ refer to the iteration counts. *L* stands for the number of neighborhoods, and *T* denote the iterations in the VNS.

As shown in Table 6, we have compared the computational complexity of each algorithm. The QGPV exhibits the highest computational complexity, which can be attributed to its two-stage optimization mechanism. In this framework, the total computational cost can be regarded as the sum of the complexities of QGA and QPSO-VNS. However, the simulation results show that the overall computation time of QGPV remains within an acceptable range, and it can still obtain the optimal solution within the same short runtime, outperforming the other four algorithms. This demonstrates that the increased computational complexity does not significantly affect the efficiency of the proposed algorithm.

**Figure 10:**
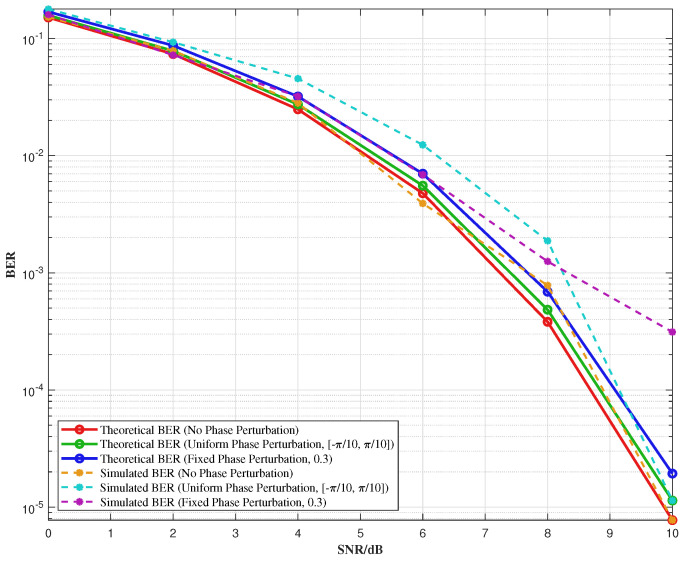
Comparison results of BER under no phase perturbation, uniform phase perturbation and fixed phase perturbation.

Figure 10 illustrates the simulation results showing the variation in BER concerning the SNR at the decision-making input terminal. The BER is obtained by the statistics of $10^{2}$ tests, with the phase perturbation range set to $\left[-\pi /10,\pi /10\right]$. The figure shows that the BER increases slightly after introducing phase perturbation. The minor discrepancy between the simulation results and the theoretical values can be attributed to the finite length of the modulated binary sequence and the limited number of simulation trials. These factors introduce small statistical variations, which cause the simulation BER to deviate slightly from the theoretical predictions. Nevertheless, the observed trends remain consistent with the theoretical analysis, and the results offer useful insights into the impact of phase perturbation on system performance.

**Figure 11:**
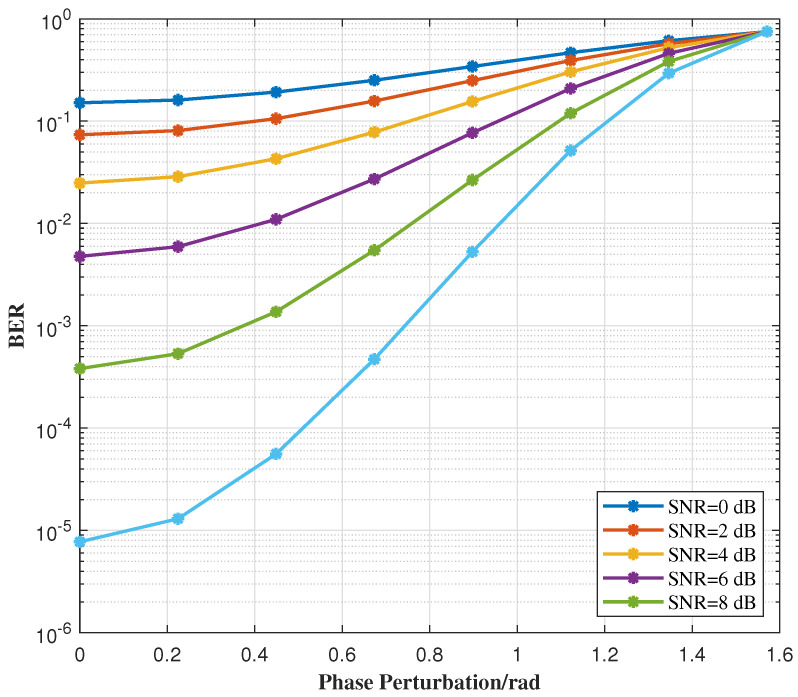
BER variation with different phase perturbations.

Figure 11 illustrates the variation in BER with phase perturbation under different SNR conditions. Since the JSAC signal is based on the QPSK modulation scheme, its phase perturbation range is defined as [0,π/2]. If the phase perturbation exceeds π/2, phase overlap occurs, significantly increasing the BER. As the figure shows, the higher the SNR, the less impact phase perturbation has on the BER. Additionally, for a fixed SNR, the BER increases as the phase perturbation range widens. These results highlight the trade-off between phase perturbation and system performance, emphasizing the importance of selecting an appropriate phase perturbation range to balance signal integrity and system requirements in practical applications. Therefore, careful consideration of the phase perturbation range is crucial for optimizing the performance of JSAC systems.

## 6. Conclusions

In this paper, we constructed an MIMO-OFDM JSAC system and focused on the generation and optimization of its transmit signals. Based on this system, a novel JSAC signal model was proposed. In particular, employing LFM as the carrier waveform significantly narrows the main lobe of the autocorrelation function, thereby improving the practical applicability of the waveform. To ensure superior orthogonality and mitigate interference, OFDM was utilized to allocate distinct frequency bands to each signal, effectively minimizing inter-channel interference. In addition, phase perturbation was introduced to increase the degrees of freedom in waveform optimization. This approach can also be extended to higher-order phase modulations, where phase perturbation can be applied to further enhance signal diversity and system adaptability. However, it should be noted that the allowable range of phase perturbation varies with the modulation type. When the perturbation range exceeds the phase decision threshold, BER increases significantly, which reduces the practical applicability of the system. Therefore, for higher-order modulation schemes, the phase perturbation must be carefully controlled to achieve a balance between optimization flexibility and communication reliability. To address the problem of high sidelobe levels in the autocorrelation function of JSAC signals, we formulated an optimization model and theoretically demonstrated that the problem is non-convex and NP-hard. Accordingly, a two-stage hybrid optimization algorithm, QGPV, was developed. In this framework, a multi-point exchange strategy was incorporated into VNS to expand its search space and strengthen its exploration capability. Furthermore, we analyzed the performance of the proposed algorithm under different sequence lengths and investigated the impact of phase perturbation on the BER.

The simulation results verified the effectiveness of the proposed waveform and optimization method. The comparative results showed that the proposed approach achieves significant advantages in suppressing the PSL while maintaining acceptable computational cost. The optimized waveform further alleviates the trade-off between system performance and algorithmic practicality, providing valuable insights for the sustainable development of JSAC technology. Future work will focus on adaptive JSAC waveform design, advanced receiver processing, resource allocation, and multi-task integration, with the goal of further enhancing the performance and practicality of JSAC systems in complex electromagnetic environments. 

## Figures and Tables

**Table 1:** The rotation angle and direction adjustment strategy of the quantum rotation gate mutation.

$g_{\mathrm{ij}}$	$b_{i}$	$\mathrm{fit}\left(g_{j}\right)\geq \mathrm{fit}\left(b\right)$	$\Delta \eta_{\mathrm{ij}}$	$e(\alpha_{\mathrm{ij}}\beta_{\mathrm{ij}})$			
				$\alpha_{\mathrm{ij}}\beta_{\mathrm{ij}}>0$	$\alpha_{\mathrm{ij}}\beta_{\mathrm{ij}}<0$	$\alpha_{\mathrm{ij}}=0$	$\beta_{\mathrm{ij}}=0$
0	0	False	0	0	0	0	0
0	0	True	0	0	0	0	0
0	1	False	0	0	0	0	0
0	1	True	0.01π	−1	+1	±1	0
1	0	False	0.01π	−1	+1	±1	0
1	0	True	0.01π	+1	−1	0	±1
1	1	False	0.01π	+1	−1	0	±1
1	1	True	0.01π	+1	−1	0	±1

**Table 2:** Comparison of QGPV under different length N conditions with the global optimal solution (dB).

	N=4	N=8	N=16
QGPV	−12.0	−16.8	−17.0
Global Optimization	−12.0	−18.06	−17.8

**Table 3:** PSL optimization results for 4 signals using QPSO, QGA, QGA-QPSO, QPSO-VNS, and QGPV.

	Signal 1 (dB)	Signal 2 (dB)	Signal 3 (dB)	Signal 4 (dB)
Original	−15.3	−12.6	−13.9	−14.5
QPSO	−15.9	−13.6	−14.5	−15.4
QGA	−17.6	−17.0	−17.1	−17.6
QGA-QPSO	−19.4	−18.8	−18.5	−19.4
QPSO-VNS	−16.7	−14.1	−15.4	−16.8
QGPV	−21.2	−20.3	−20.5	−20.8

**Table 4:** Average running time under 5 iterations.

Algorithm	Average PSL (dB)	Average Running Time (s)
QPSO	−14.0	0.02
QGA	−17.6	0.01
QGA-QPSO	−18.9	0.03
QPSO-VNS	−15.0	0.11
QGPV	−20.5	0.10

**Table 5:** PSL optimization performance comparison of algorithms under the same running time (0.1 s).

Algorithm	Average PSL (dB)	Iterations
QPSO	−14.9	28
QGA	−18.0	43
QGA-QPSO	−19.2	37
QPSO-VNS	−15.6	5
QGPV	−20.3	5

**Table 6:** Computational complexity comparison of algorithms.

Algorithm	Computational Complexity
QPSO	$O(I_{g}\cdot J_{g}\cdot K_{g})$
QGA	$O(I_{p}\cdot J_{p}\cdot K_{p})$
QGA-QPSO	$O\left(I_{g}\cdot J_{g}\cdot K_{g}\right)+O\left(I_{p}\cdot J_{p}\cdot K_{p}\right)$
QPSO-VNS	$O(\left(I_{g}\cdot J_{g}+L\cdot T\right)\cdot K_{g})$
QGPV	$O\left(I_{g}\cdot J_{g}\cdot K_{g}\right)+O\left(\left(I_{g}\cdot J_{g}+L\cdot T\right)\cdot K_{g}\right)$

## Data Availability

The data presented in this study are available on request from the corresponding author. The data are not publicly available due to privacy.
